# *Weissella confusa* F213 ameliorated inflammation and maintained intestinal mucosa integrity in chemically induced colitis rats

**DOI:** 10.1186/s13104-023-06456-2

**Published:** 2023-08-22

**Authors:** I Nengah Sujaya, Ida Ayu Gde Wahyudevi Dharmika, Gede Ngurah Rsi Suwardana, I Ketut Mariadi, I Gusti Kamasan Nyoman Arijana, Ida Bagus Oka Winaya, Komang Ayu Nocianitri, Yan Ramona, Ni Nengah Dwi Fatmawati

**Affiliations:** 1 School of Public Health, Faculty of Medicine, Udayana University, Bali, Indonesia; 2 Department of Microbiology, Faculty of Medicine, Udayana University, Bali, Indonesia; 3 Division Gastroenterohepatology, Department of Internal Medicine, Udayana University/Professor Dr. I.G.N.G. Ngoerah Hospital, Denpasar, Bali, Indonesia; 4 Department of Histology, Faculty of Medicine, Udayana University, Bali, Indonesia; 5 Pathology Anatomy Laboratory, Faculty of Veterinary, Udayana University, Bali, Indonesia; 6 School of Food Technology, Faculty of Agricultural Technology, Udayana University, Bali, Indonesia; 7 School of Biology, Faculty of Mathematics and Natural Sciences, Udayana University, Bali, Indonesia

**Keywords:** Probiotics, *Weissella confusa* F213, Colitis, Inflammation, Mucosa integrity

## Abstract

**Objective:**

This study was performed to investigate the potential effects of *Weissella confusa* F213 (WCF213) on chemically-induced colitis rats. Twelve male Wistar rats were divided into three groups: T1 (saline sterile), T2 (2.5% dextran sulfate sodium (DSS)- for 7 days), and T3 (WCF213 for 14 days, continued with 2.5% DSS for 7 days). The disease activity index (DAI) was monitored. After sacrificing the rats, the colon was collected for length measurement, local TNF-α level, HE staining for histology, and ZO-1 expression by using immunohistochemistry.

**Results:**

WCF213 administration prevented weight loss and haematochezia, maintained average colon length and alleviated the clinical symptom of colitis, such as diarrhoea, albeit statistically non-significant (*p* < 0.05) compared with the T2 group. The histopathology of WCF213-treated colitis rats showed better architecture and less inflammatory cell infiltration into colon tissue. WCF213 significantly maintained the expression of ZO-1 in the mucosa (*p* < 0.001) and markedly reduced mucosal TNF-α concentration (*p* < 0.001) compared with the DSS group. Hence, these findings suggested that WCF213 attenuated clinical symptoms and inflammation and maintained mucosal integrity in DSS-induced colitis in vivo.

**Supplementary Information:**

The online version contains supplementary material available at 10.1186/s13104-023-06456-2.

## Introduction

Inflammatory bowel disease (IBD) is a chronic inflammation of the gastrointestinal tract, represented by Crohn’s disease (CD) and ulcerative colitis (UC) [1, 2]. IBD is characterized by abdominal pain, diarrhoea (also with bleeding), fever, weakness, fatigue, weight loss, and malnutrition [3, 4]. The incidence and prevalence of IBD among Asia tend to increase recently [5]. The specific aetiology of IBD is still unknown. Multifactorial causes, such as genetics, aberrant immune response, disturbance of the intestinal barrier, and gastrointestinal microbiome dysbiosis, have been suggested to play vital roles in the occurrence and progression of IBD [6–8]. Disturbance of intestinal tight junctions (TJs) leads to increased permeability, thus allowing bacterial (or its metabolites) and harmful substances leakage into the bloodstream to be found in IBD [9]. Therefore, maintaining mucosal integrity is essential in IBD management. One IBD treatment approach is using probiotics [10, 11]. Probiotics are widely known to have potential benefits for treating IBD [12]. Probiotics are defined as live microorganisms that exert beneficial effects on the host if given in adequate amounts [13, 14]. The immunomodulatory property of probiotics is proposed due to their ability to maintain mucosal barrier function or enhancement of regulatory T (T_reg_) cell activity in modulating proinflammatory (TNF-α and IL-1β) and anti-inflammatory cytokines (IL-10) [15]. Furthermore, probiotics can strengthen tight junctions of the gut mucosa barrier in vitro and in vivo [4, 16, 17]*. Lactobacillus* spp. and *Bifidobacterium* spp. are the most frequently utilized lactic acid bacteria (LAB) in probiotic products [18]. With recent technological advances in culturing methodologies, better tools to edit and modify bacterial genomes, and the reduced cost of genome and metagenome sequencing [19, 20], many studies have been conducted to explore the next-generation probiotics (NGPs) species, including *Akkermansia muciniphila* and *Faecalibacterium prausnitzii* [21, 22]*.* Several species of *Weissella* have also been reported as potential new probiotic candidates with many prospective applications [23]. In clinical practice, the efficacy of probiotics highly depends on the strain and dosage [24]. Our group has isolated a promising strain of *Weissella confusa* F213 (WCF213) from human origin [25]. Both in vitro and in vivo studies have been investigated to confirm the safety of this strain [25–28]. In a previous study, WCF213 successfully maintained tight junctions in in vitro models of IBD [16]. This interesting finding urges further investigation in animal models. The current study explored the functional effect of WCF213 on chemically-induced colitis rats.

## Methods

### Bacterial cells preparation

*Weissella confusa* F213 (WCF213) (The Integrated Laboratory of Bioscience and Biotechnology, Udayana University) was grown in de Mann Rogosa Sharpe (MRS) broth (Oxoid, Basingstoke, UK) at 37 °C anaerobically for 18 h. The bacterial pellets were harvested through centrifugation at 13,000 rpm at 4 °C for 5 min. The bacterial suspension was prepared in normal saline (1 × 10^9^ CFU/ml WCF213) and used for the probiotic-treated group.

### Reagent for membrane disruption

Dextran Sulfate Sodium/DSS with a molecular weight of 36–50 kDa (MP Biomedicals, OH, USA) was used for inducing colitis in rats.

### Animal models

A total of 12 male Wistar rats (approximately 100 g each) were used in this study. The rats were maintained at animal housing facilities that apply the animal welfare principles (24 ± 1 °C; 12-h light/dark cycle; 55% humidity, and had access to rodent standard food and water ad libitum) (Animal Laboratory Centre of The Integrated Biomedical Laboratory, Faculty of Medicine, Udayana University, Bali, Indonesia).

### The effect of *Weissella confusa* F213 on DSS-induced animal model experiments

Rats were allocated to one of three experimental groups (n = 4 each): Group 1 (T1), which received sterile saline; Group 2 (T2), which received 2.5% DSS for 7 days; and Group 3, which received WCF213 for 14 days and then continued with 2.5% DSS for 7 days. After 1 week of acclimatization, the rats were treated based on designated protocols. The probiotics were administered through oral gavage, while DSS was administered through drinking water ad libitum. The colitis severity was evaluated using Disease Activity Index (DAI) score with modification for the perianal bleeding parameter [30].

### Colon collection

A combination of ketamine and xylazine was used for rats’ anaesthesia, and then they were humanely euthanized by cervical dislocation. The colons of the rats were then harvested, and the length was measured. The colon was used for histopathological examination, Zonula Occludens (ZO)-1 immunohistochemistry (IHC) and TNF-α ELISA. The colon used for histopathological and immunohistochemistry assays was fixed in 10% buffered formalin solution overnight before proceeding to the assays, while the colon for ELISA was kept at − 80 °C until performing the assay.

### Histopathological examination

A histopathological examination was performed to examine the histological structure of the colon. The organ tissues were embedded in paraffin wax for 3 h after 10% phosphate-buffered formalin (Merck, Germany) fixation and overnight dehydration. The 5 μm-thick sections (Jung Leica 820 Histocut, Leica, Germany) were fixed on a glass slide, dried at 60 °C, and stained with haematoxylin and eosin (Leica, Germany). The colon structure was observed under a microscope (Olympus CX 41, Olympus, Japan) and captured with a camera (OptilabPro, Miconos, Indonesia).

### Zonula occludens-1 (ZO-1) immunohistochemistry

Immunohistochemistry (IHC) of the Zonula Occludens-1 (ZO-1) was performed as previously mentioned with modifications [31, 32]. Briefly, 5 μm-sections of colon tissue were deparaffinized and rehydrated. The slides were heated in an incubator for 60 min at 90 °C, immersed in sodium citrate pH 6.0, followed by blocking (Peroxide Block, SCYTEK Laboratories Inc., USA). Rabbit anti-rat ZO-1 polyclonal antibody (1/100 dilution) (Invitrogen, USA) were applied to the slides and incubated overnight. After rinsing the primary antibody, the slides were flooded with a secondary antibody, CRF Anti-Polyvalent HRP (SCYTEK Laboratories Inc., USA). Chromogenic detection was carried out using DAB chromogen (SCYTEK Laboratories Inc., USA), and the counterstaining was conducted using haematoxylin Meyer (Leica, Germany). The IHC results were examined under a microscope (Olympus CX 41, Olympus, Japan) and photographed (OptilabPro, Miconos, Indonesia). The cells expressing ZO-1 (400-fold magnification) were shown as brown on the edges of the epithelial cells. Images were randomly observed from three fields of view, and the average of the cells expressing ZO-1 was calculated.

### Determination of colonic TNF-α level

Colon mucosal TNF-α levels were measured by ELISA (Elabscience^®^, USA) based on the manufacturer’s instructions. Frozen colonic samples were homogenized in 1 mL of cold PBS and centrifuged (15,000×*g*, 10 min). The colonic supernatant TNF-α concentration was measured at 450 nm (ELISA reader 270, bioMérieux, France). Data were expressed as picogram/millilitre. All measurements were performed in duplicate.

### Statistical analysis

The experiments were done in duplicate unless otherwise indicated. All statistical analysis was calculated using IBM SPSS program (version 25.0, Chicago, USA). Data are presented as the means ± standard errors of the means (SEM) for each group. For comparative analysis, multiple comparisons were calculated using one-way ANOVA and Tukey’s post hoc test. *P* values < 0.05 were considered statistically significant.

## Results

### *Weissella confusa* F213 attenuated colitis clinical symptom severity

DAI scoring was used for evaluating the WCF213 effect on colitis severity [30]. The study showed that WCF213 reduced the DAI score parameters, such as reductions in weight loss, diarrhoea, and perianal bleeding score (Additional file 1). Specifically, treatment with WCF213 in colitis animals effectively attenuated DSS-induced weight loss compared to DSS-treated animals (*p* = 0.02). Furthermore, this probiotic could effectively reduce the clinical symptoms of diarrhoea in colitis animals (*p* = 0.000). Moreover, WCF213 could also alleviate perianal bleeding and maintained colon length, although it could not achieve significant results (*p* = 0.246 and *p* = 0.063, respectively).

### *Weissella confusa* F213 prevented histological damage to the colon in colitis animal models

**Fig. 1:**
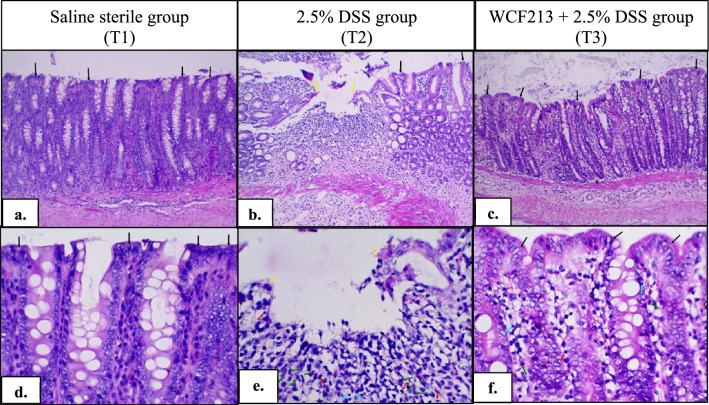
Histopathology of the colon. Histologic examination of T1 (**a**) showed no erosive lesions or extensive inflammatory cell infiltration in the colon tissue (100 × magnifications). Very few immune cells were observed in this group (**d**) (400 × magnifications). The histologic examination of the T3 group showed no erosive lesions and no extensive inflammatory cell infiltration in the colon tissue (**c**) (100 × magnifications); however, more immune cells were observed (**f**) (400-fold magnification). Although the colon tissue of the colitis rat obtained probiotics showed some inflammatory lesions, the severity of inflammation was less than that in the DSS rat (**b** and **e**). (yellow arrow: discontinued villus; black arrow: intact villus, red arrow: macrophage; green arrow: neutrophil; blue arrow: lymphocyte)

Damage to the colon mucosa in DSS-induced colitis can be displayed as infiltration of inflammatory cells into the colon mucosa and erosive lesions of the colon mucosa. The histopathological examination revealed no erosive lesions or inflammatory cell infiltration in the colon tissue, and very few immune cells were observed in normal colon mucosa (Fig. 1a, d). In addition, the colon mucosa of the WCF213-treated colitis group showed fewer erosive and inflammatory lesions, maintained crypt structures (Fig. 1c), and less infiltration of inflammatory cells into the colon mucosa (Fig. 1f) than the DSS-treated group (Fig. 1b, e).

### *Weissella confusa* F213 improved mucosal barrier integrity in DSS-induced colitis

**Fig. 2:**
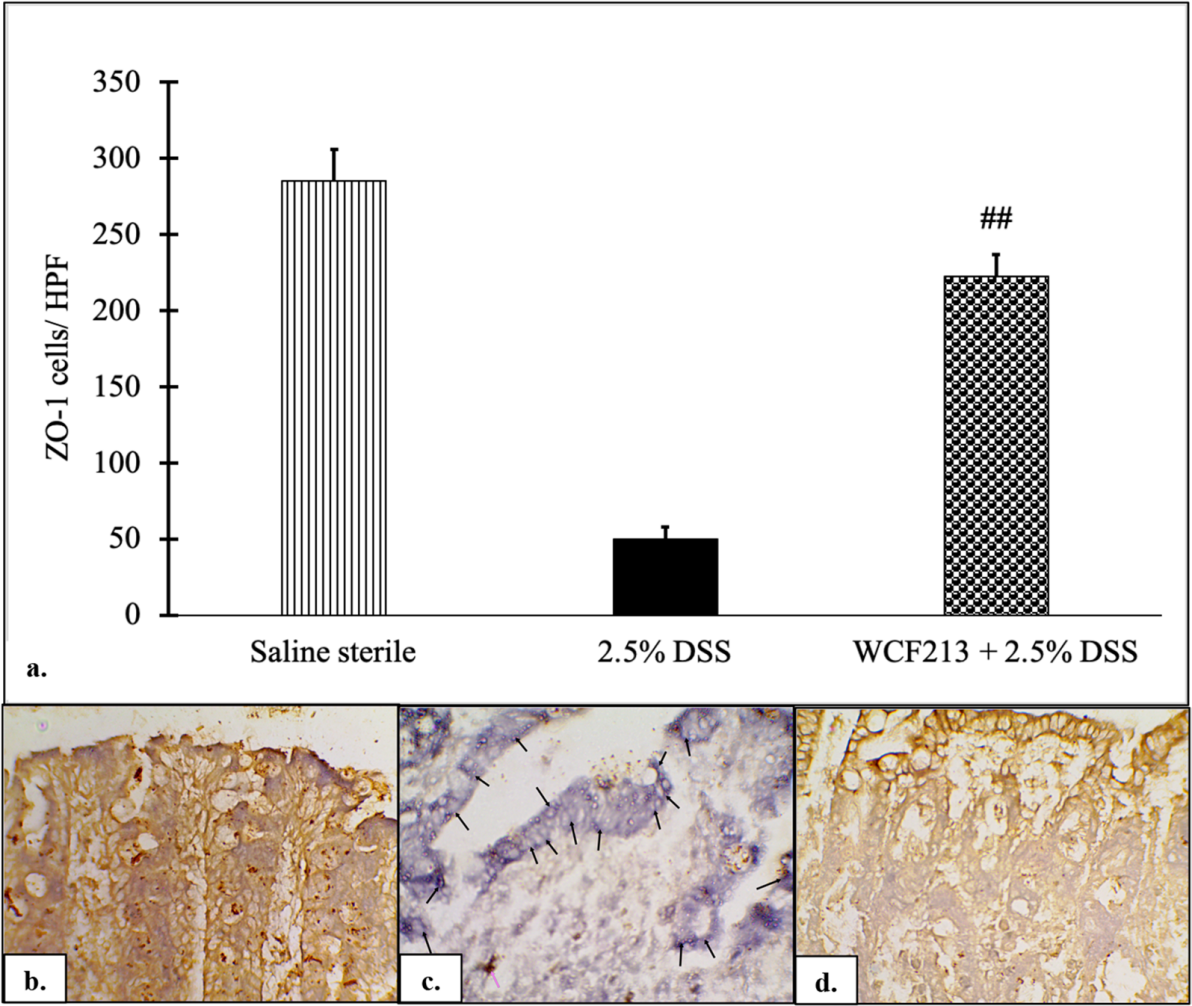
The expression of ZO-1 protein was evaluated by IHC in colon samples. WCF213 significantly maintained ZO-1 expression of tight junction (*p* = 0.000) (**a**). Moreover, ZO-1 protein expression persisted in normal and WCF213 + 2.5% DSS groups (**b**, **d**) as compared with the DSS-only group (**c**) (violet arrow: ZO-1 expressed in brown colour on the cellular membrane; black arrow: lack of brown colour as lack of ZO-1 expressed on the cellular membrane). Data expressed as mean ± SEM (n = 4). (^##^*p* < 0.001 vs. DSS group). IHC results were observed at 400-fold magnification. All observation was performed in triplicate

Tight junctions (TJs) proteins act as a selective barrier that maintains the integrity of intestinal cells. TJ protein disruption leads to increased mucosal permeability [33, 34]. One TJ protein is ZO-1. This study showed that WCF213 treatment significantly maintained ZO-1 protein in DSS-induced colitis rats (*p* = 0.000; Fig. 2, Additional file 1).

### *Weissella confusa* F213 reduced colonic proinflammatory cytokine levels

**Fig. 3:**
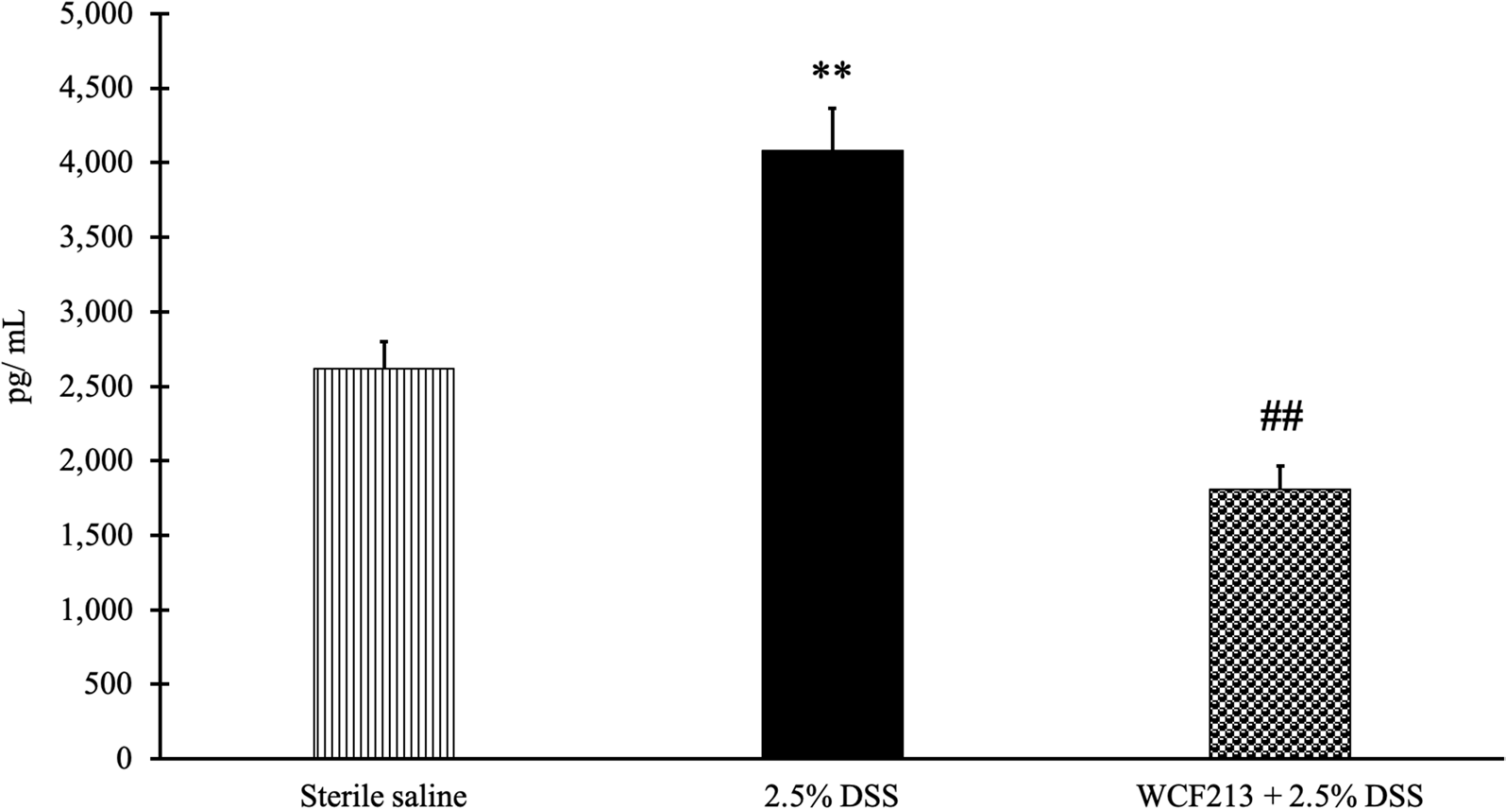
Colonic TNF-α level measurement. This study showed that WCF213 could alleviate colon mucosa TNF-α concentration (T3) compared to the DSS group. Data expressed as mean ± SEM (n = 4). **p < 0.01 vs. sterile saline (T1) group; ^##^*p* < 0.01 vs. DSS group (T2)

Inflammation can mediate tight junction (TJ) disruption, leading to colitis. This study found that WCF213 significantly decreased the colon mucosa TNF-α concentration (*p* = 0.000, Fig. 3, Additional file 1).

## Discussion

Numerous studies have been conducted to reduce clinical symptoms, inflammation, and mucosal integrity in IBD using probiotics [11, 35, 36]. However, since the effect of probiotics is strain-dependent, many researchers have investigated potential probiotic strains. In a previous study, our group found that the WCF213 strain maintained mucosal integrity in an in vitro model of IBD [16]. The current experiment discovered that WCF213 diminished the clinical symptoms of colitis, as shown by the decrease in weight reduction, DAI score, and colon length. This study was in concurrence with an experiment led by Wang et al. (2021) that revealed that *Bifidobacterium lactis* XLTG1 alleviated the symptoms of colitis in animal models, as confirmed by the reduction in colon length, weight loss, DAI score, and spleen index [6]. Likewise, in this study, histological analysis reported that WCF213 effectively maintained the colon mucosa structure. Shin et al. (2020) reported a similar result, in which probiotic treatment in DSS-induced colitis mice successfully improved the histological structure of the colon [37]. The findings in this study suggested that WCF213 reduced the severity of colitis clinical symptoms in DSS-treated rats and maintained the histological structure of the intestinal epithelium.

Disruption of TJ proteins increases mucosal permeability, which could be an aetiological factor for gastrointestinal disorders, including IBD [38–40]. ZO-1 protein is necessary for the initial assembly of TJ proteins into the cells and connecting adjacent cells. TJ function may be compromised by ZO-1 protein disruption or absence [41]. Shin et al. (2020) observed that the expression of ZO-1 protein was maintained in *L. brevis* Bmb6-treated colitis mice compared with DSS colitis mice [37]. The current study found that WCF213 significantly maintained ZO-1 protein expression in WCF213-treated colitis rats, supporting that WCF213 maintained mucosal barrier integrity.

Abnormal inflammatory reactions occur in IBD. The proinflammatory cytokine TNF-α induces inflammation, leading to TJ disruption and elevation of mucosal permeability [12, 40]. In this study, colonic TNF-α in the DSS group treated with WCF213 was significantly reduced. A previous study showed a similar situation where *L. brevis* Bmb6 reduced TNF-α gene expression [37]. In addition, another study discovered that supplementation with *L. plantarum* HNU082 effectively downregulated proinflammatory cytokines in DSS-induced mice [42]. The findings in the present study suggested that WCF213 attenuated inflammation mediated by TNF-α, therefore, reduced TJ disturbance.

In this study, WCF213 successfully reduced the severity of clinical symptoms of colitis, maintained mucosa integrity, and reduced inflammation in vivo; therefore, these findings supported that WCF213 is promising for supplementation or an alternative strategy in IBD management, which needs to be further investigated in humans.

### Limitations

Depletion of mucus barrier as a result of goblet cells numbers reduction has been associated with IBD [43]. However, we did not specifically evaluate the number of goblet cells in this study; thus, the role of WCF213 in maintaining the mucus layer needs to be further elucidated. Since dysbiosis is related to IBD, further investigation is expected to determine the effect of WCF213 in gastrointestinal microbiota modulation and short-chain fatty acid (SCFA) production in DSS-induced animal models.

### Supplementary Information

**Additional file 1**: Measurement Results and Statistical Analysis of Disease Activity Index (DAI), Colon Length, The Expression of Zonula Occludens-1 Protein, and Colon Mucosa TNF-$\varvec{ \alpha}$ Concentration.

## Data Availability

All data are included in this published article [and its supplementary information files].

## Abbreviations

WCF213: *Weissella confusa* F213
DSS: Dextran sulfate sodium
DAI: Disease activity index
IHC: Immunohistochemistry
ZO-1: Zona Occludens-1
IBD: Inflammatory bowel disease
UC: Ulcerative colitis
CD: Crohn’s disease
TJs: Tight junctions
MRS: De Mann, Rogosa, and Sharpe
SCFA: Short-chain fatty acid
