# Supplementary material for: Weissella confusa F213 ameliorated inflammation and maintained intestinal mucosa integrity in chemically induced colitis rats
Source: BMC Res Notes. 2023 Aug 22;16:178. doi: 10.1186/s13104-023-06456-2 (PMC10463849; doi:10.1186/s13104-023-06456-2)
Supplement: Supplementary file 1 — Additional file 1: Measurement Results and Statistical Analysis of Disease Activity Index (DAI), Colon Length, The Expression of Zonula Occludens-1 Protein, and Colon Mucosa TNF-$\varvec{ \alpha}$α Concentration. [file 13104_2023_6456_MOESM1_ESM.docx]

**Supplementary Data**

Table 1 Disease Activity Index (DAI) and Colon Length Measurement Results*

|  | Weight Loss Score | Stool Consistency Score | Perianal bleeding | Colon Length |
| --- | --- | --- | --- | --- |
| Sterile saline | 0.25 ± 0.25 | 0.00 ± 0.00 | 0.00 ± 0.00 | 10.50 ± 0.89 |
| 2.5% DSS | 3.50 ± 0.29 | 4.00 ± 0.00 | 3.00 ± 1.00 | 6.28 ± 0.36 |
| WCF213 + 2.5% DSS | 2.00 ± 0.00 | 1.00 ± 0.58 | 1.00 ± 1.00 | 8.43 ± 0.25 |

*Mean ± SEM

Table 2 Statistical Analysis of Disease Activity Index (DAI) and Colon Length

|  | | | Sig. | 95% Confidence Interval | |
| --- | --- | --- | --- | --- | --- |
|  |  |  |  | Lower Bound | Upper Bound |
| Weight loss score | Sterile saline | WCF213+2.5% DSS | **0.001*** | -2.6206 | -0.8794 |
|  |  | 2.5% DSS | **0.000**** | -4.1206 | -2.3794 |
|  | 2.5% DSS | Sterile saline | **0.000**** | 2.3794 | 4.1206 |
|  |  | WCF213+2.5% DSS | **0.002*** | 0.6294 | 2.3706 |
|  | WCF213+2.5% DSS | Sterile saline | **0.001*** | 0.8794 | 2.6206 |
|  |  | 2.5% DSS | **0.002*** | -2.3706 | -0.6294 |
| Stool Consistency Score | Sterile saline | WCF213+2.5% DSS | 0.140 | -2.3162 | 0.3162 |
|  |  | 2.5% DSS | **0.000**** | -5.3162 | -2.6838 |
|  | 2.5% DSS | Sterile saline | **0.000**** | 2.6838 | 5.3162 |
|  |  | WCF213+2.5% DSS | **0.000**** | 1.6838 | 4.3162 |
|  | WCF213+2.5% DSS | Sterile saline | 0.140 | -0.3162 | 2.3162 |
|  |  | 2.5% DSS | **0.000**** | -4.3162 | -1.6838 |
| Perianal bleeding score | Sterile saline | WCF213+2.5% DSS | 0.674 | -4.2239 | 2.2239 |
|  |  | 2.5% DSS | 0.068 | -6.2239 | 0.2239 |
|  | 2.5% DSS | Sterile saline | 0.068 | -0.2239 | 6.2239 |
|  |  | WCF213+2.5% DSS | 0.246 | -1.2239 | 5.2239 |
|  | WCF213+2.5% DSS | Sterile saline | 0.674 | -2.2239 | 4.2239 |
|  |  | 2.5% DSS | 0.246 | -5.2239 | 1.2239 |
| Colon Length (cm) | Sterile saline | WCF213+2.5% DSS | 0.073 | -0.1947 | 4.3447 |
|  |  | 2.5% DSS | **0.001*** | 1.9553 | 6.4947 |
|  | 2.5% DSS | Sterile saline | **0.001*** | -6.4947 | -1.9553 |
|  |  | WCF213+2.5% DSS | 0.063 | -4.4197 | 0.1197 |
|  | WCF213+2.5% DSS | Sterile saline | 0.073 | -4.3447 | 0.1947 |
|  |  | 2.5% DSS | 0.063 | -0.1197 | 4.4197 |

Table 3 The expression of Zonula Occludens-1 protein*

|  | ZO-1 |
| --- | --- |
| Sterile saline | 279.08 ± 21.77 |
| 2.5% DSS | 46.15 ± 6.67 |
| WCF213 + 2.5% DSS | 238.15 ± 5.94 |

*Mean ± SEM

Table 4 Statistical Analysis of the expression of Zonula Occludens-1 protein

|  | | Sig. | 95% Confidence Interval | |
| --- | --- | --- | --- | --- |
|  |  |  | Lower Bound | Upper Bound |
| Sterile Saline | WCF213 + DSS 2.5% | 0.138 | -12.7199 | 94.5699 |
|  | DSS 2.5% | **0.000**** | 179.2801 | 286.5699 |
| WCF213 + DSS 2.5% | Sterile Saline | 0.138 | -94.5699 | 12.7199 |
|  | DSS 2.5% | **0.000**** | 138.3551 | 245.6449 |
| DSS 2.5% | Sterile Saline | 0.000** | -286.5699 | -179.2801 |
|  | WCF213 + DSS 2.5% | **0.000**** | -245.6449 | -138.3551 |

Table 5 Colon Mucosa TNF-𝝰 Concentration*

|  | TNF-𝝰 (pg/mL) |
| --- | --- |
| Sterile saline | 2621.94 ± 177.82 |
| 2.5% DSS | 4077.50 ± 285.78 |
| WCF213 + 2.5% DSS | 1811.48 ± 156.67 |

*Mean ± SEM

Table 6 Statistical Analysis of Colon Mucosa TNF-𝝰 Concentration

|  | | Sig. | 95% Confidence Interval | |
| --- | --- | --- | --- | --- |
|  |  |  | Lower Bound | Upper Bound |
| Sterile saline | WCF213+2.5% DSS | 0.050 | -1.1248 | 1622.0507 |
|  | 2.5% DSS | **0.000**** | -2206.9398 | -704.1713 |
| WCF213+2.5% DSS | Sterile saline | 0.050 | -1622.0507 | 1.1248 |
|  | 2.5% DSS | **0.000**** | -3077.6063 | -1454.4308 |
| 2.5% DSS | Sterile saline | **0.000**** | 704.1713 | 2206.9398 |
|  | WCF213+2.5% DSS | **0.000**** | 1454.4308 | 3077.6063 |
